# Supplementary material for: SPInDel Analysis of the Non-Coding Regions of cpDNA as a More Useful Tool for the Identification of Rye (Poaceae: Secale) Species
Source: Int J Mol Sci. 2020 Dec 10;21(24):9421. doi: 10.3390/ijms21249421 (PMC7762986; doi:10.3390/ijms21249421)
Supplement: Supplementary file 1 [file ijms-21-09421-s001.pdf]

International Journal of
Molecular Sciences

MDPI

Supplementary Tables

Table 1. The list of species, along with the accession numbers for each sample.

No.	Species	Origin	NCBI Genbank				Accession number	Type[a]	Life Cycle[a]
			[b]*cpDNA-regions*		[c]*mtDNA-regions*				
**1**	*Secale cereale* ssp. *afghanicum*	Armenia	1	MH893827	5	MH893967	PI 618662	We	A
			2	MH893862	6	MH894002			
			3	MH893897	7	MH894037			
			4	MH893932	8	MH894072			
					9	MH894107			
					10	MH894142			
**2**	*Secale cereale* ssp. *ancestrale*	Japan	1	MH893828	5	MH893968	CI SE 107	We	A
			2	MH893863	6	MH894003			
			3	MH893898	7	MH894038			
			4	MH893933	8	MH894073			
					9	MH894108			
					10	MH894143			

**3**	Russia	1	MH893829	5	MH893969	PI 445975
		2	MH893864	6	MH894004	
		3	MH893899	7	MH894039	
		4	MH893934	8	MH894074	
				9	MH894109	
				10	MH894144	
**4**	Turkey	1	MH893830	5	MH893970	PI 618666
		2	MH893865	6	MH894005	
		3	MH893900	7	MH894040	
		4	MH893935	8	MH894075	
				9	MH894110	
				10	MH894145	
**5**	USA	1	MH893831	5	MH893971	PI 445976
		2	MH893866	6	MH894006	
		3	MH893901	7	MH894041	
		4	MH893936	8	MH894076	

					9	MH894111			
					10	MH894146			
**6**	*Secale cereale* ssp. *cereale*	Canada	1	MH893832	5	MH893972	PI 590948	C	A
			2	MH893867	6	MH894007			
			3	MH893902	7	MH894042			
			4	MH893937	8	MH894077			
					9	MH894112			
					10	MH894147			
**7**		Pakistan	1	MH893833	5	MH893973	PI 561809		
			2	MH893868	6	MH894008			
			3	MH893903	7	MH894043			
			4	MH893938	8	MH894078			
					9	MH894113			
					10	MH894148			
**8**		USA	1	MH893834	5	MH893974	PI 628642		
			2	MH893869	6	MH894009			

			3	MH893904	7	MH894044			
			4	MH893939	8	MH894079			
					9	MH894114			
					10	MH894149			
**9**		Tajikistan	1	MH893835	5	MH893975	PI 639383		
			2	MH893870	6	MH894010			
			3	MH893905	7	MH894045			
			4	MH893940	8	MH894080			
					9	MH894115			
					10	MH894150			
**10**	*Secale cereale* ssp. *segetale*	Azerbaijan	1	MH893836	5	MH893976	PI 267102	We	A
			2	MH893871	6	MH894011			
			3	MH893906	7	MH894046			
			4	MH893941	8	MH894081			
					9	MH894116			
					10	MH894151			

**11**	Russia	1	MH893837	5	MH893977	PI 283982
		2	MH893871	6	MH894012	
		3	MH893907	7	MH894047	
		4	MH893942	8	MH894082	
				9	MH894117	
				10	MH894152	
**12**	Kazakhstan	1	MH893838	5	MH893978	PI 326286
		2	MH893872	6	MH894013	
		3	MH893908	7	MH894048	
		4	MH893943	8	MH894083	
				9	MH894118	
				10	MH8941453	
**13**	Turkey	1	MH893839	5	MH893979	PI 618673
		2	MH893873	6	MH894014	
		3	MH893909	7	MH894049	
		4	MH893944	8	MH894084	

					9	MH894119			
					10	MH894154			
**14**	*Secale cereale* ssp. *dighoricum*	Sweden	1	MH893840	5	MH893980	PI 618667	We	A
			2	MH893874	6	MH894015			
			3	MH893910	7	MH894050			
			4	MH893945	8	MH894085			
					9	MH894120			
					10	MH8941455			
**15**		Russia	1	MH893841	5	MH893981	PI 618668		
			2	MH893875	6	MH894016			
			3	MH893911	7	MH894051			
			4	MH893946	8	MH894086			
					9	MH894121			
					10	MH8941456			
**16**	*Secale cereale* ssp.	Turkey	1	MH893842	5	MH893982	PI 618669	We	A
			2	MH893876	6	MH894017			

			3	MH893912	7	MH894052			
			4	MH893947	8	MH894087			
					9	MH894122			
					10	MH894157			
**17**		Poland	1	MH893843	5	MH893983	124197		
			2	MH893877	6	MH894018			
			3	MH893913	7	MH894053			
			4	MH893948	8	MH894088			
					9	MH894123			
					10	MH8941458			
**18**	*Secale strictum* ssp. *africanum*	RSA	1	MH893844	5	MH893984	PI 630963	Wi	P
			2	MH893878	6	MH894019			
			3	MH893914	7	MH894054			
			4	MH893949	8	MH894089			
					9	MH894124			
					10	MH8941459			

**19**		RSA	1	MH893845	5	MH893985	PI 630964		
			2	MH893879	6	MH894020			
			3	MH893915	7	MH894056			
			4	MH893950	8	MH894090			
					9	MH894125			
					10	MH894160			
**20**	*Secale strictum* ssp. *anatolicum*	USA	1	MH893846	5	MH893986	PI 445973	Wi	P
			2	MH893880	6	MH894021			
			3	MH893916	7	MH894057			
			4	MH893951	8	MH894091			
					9	MH894126			
					10	MH894161			
**21**		Canada	1	MH893847	5	MH893987	PI 445974		
			2	MH893881	6	MH894022			
			3	MH893917	7	MH894058			
			4	MH893952	8	MH894092			

					9	MH894127			
					10	MH8941462			
**22**		Turkey	1	MH893848	5	MH893988	PI 630965		
			2	MH893882	6	MH894023			
			3	MH893918	7	MH894059			
			4	MH893953	8	MH894093			
					9	MH894128			
					10	MH894163			
**23**	*Secale strictum* ssp. *kuprijanovii*	Poland	1	MH893849	5	MH893989	PI 31328	Wi	P
			2	MH893883	6	MH894024			
			3	MH893919	7	MH894060			
			4	MH893954	8	MH894094			
					9	MH894129			
					10	MH8941464			
**24**	*Secale strict*	Poland	1	MH893850	5	MH893990	PI 630971	Wi	P

		2	MH893884	6	MH894025	
		3	MH893920	7	MH894061	
		4	MH893955	8	MH894095	
				9	MH894130	
				10	MH894165	
**25**	Hungary	1	MH893851	5	MH893991	PI 272338
		2	MH893885	6	MH894026	
		3	MH893921	7	MH894062	
		4	MH893956	8	MH894096	
				9	MH894131	
				10	MH894166	
**26**	Iraq	1	MH893852	5	MH893992	PI 253956
		2	MH893886	6	MH894027	
		3	MH893922	7	MH894063	
		4	MH893957	8	MH894097	
				9	MH894132	

					10	MH894167			
**27**	*Secale strictum* ssp. *ciliatoglume*	Poland	1	MH893853	5	MH893993	PI 630967	Wi	P
			2	MH893887	6	MH894028			
			3	MH893923	7	MH894064			
			4	MH893958	8	MH894098			
					9	MH894133			
					10	MH894168			
**28**	*Secale vavilovii*	Afghanistan	1	MH893854	5	MH893994	PI 57364	Wi	A
			2	MH893888	6	MH894029			
			3	MH893924	7	MH894065			
			4	MH893959	8	MH894099			
					9	MH894134			
					10	MH894169			
**29**		Russia	1	MH893855	5	MH893995	PI 573648		
			2	MH893889	6	MH894030			
			3	MH893925	7	MH894064			

			4	MH893960	8	MH894100				
					9	MH894135				
					10	MH894170				
**30**		Hungary	1	MH893856	5	MH893996	PI 28842			
			2	MH893890	6	MH894031				
			3	MH893926	7	MH894065				
			4	MH893961	8	MH894101				
					9	MH894136				
					10	MH894171				
**31**		Poland	1	MH893857	5	MH893997	831/96			
			2	MH893891	6	MH894032				
			3	MH893927	7	MH894066				
			4	MH893962	8	MH894102				
					9	MH894137				
					10	MH894172				
**32**	*Secale*	Russia	1	MH893858	5	MH893998		6047	Wi	P

		2	MH893892	6	MH894033	
		3	MH893928	7	MH894067	
		4	MH893963	8	MH894103	
				9	MH894138	
				10	MH894173	
**33**	Hungary	1	MH893859	5	MH893999	PI 615332
		2	MH893893	6	MH894034	
		3	MH893929	7	MH894068	
		4	MH893964	8	MH894104	
				9	MH894139	
				10	MH894174	
**34**	Bulgaria	1	MH893860	5	MH894000	PI 618675
		2	MH893894	6	MH894035	
		3	MH893930	7	MH894069	
		4	MH893965	8	MH894105	
				9	MH894140	

				10	MH894175	
**35**	Poland	1	MH893861	5	MH894001	PI 618676
		2	MH893895	6	MH894035	
		3	MH893931	7	MH894070	
		4	MH893966	8	MH894105	
				9	MH894140	
				10	MH894176	

[a] Wi- wild, We – weedy, C - cultivated, P – parennial, A – annual

[b]cp-DNA regions:1- *atpB-rbcL intergenic spacer*, 2- *trnT(UGU)-trnL5'exon intergenic spacer,* 3- *trnL(UAA) intron intergenic spacer*, 4- *trnD[tRNA-Asp(GUC)]-trnT [tRNA-Thr(GGU)]*;

[c]mt-DNA regions: 5- *nad1 exon B-nad1 exon C intron intergenic spacer*, 6-*nad4/1-2 intergenic spacer*,7-*nad4L-orf25 intergenic spacer*,8-*rps12-1/nad3-2 intergenic spacer*,9-*rps12-2/nad3-1 intergenic spacer*,10-*rrn5/rrn18-1 intergenic spacer*

Table S2. General description of standard SPInDel profiles from the *atpB-rbcL* cpDNA region.

Species	Number of Sequences (N)	Number of Conserved Regions	Number of Hypervariable Regions (n)	Average Number of Pairwise Differences ($p^{G}{}_{n}$)	Average Number of Pairwise Differences per Hypervariable Region	Number of Species-Specific Profiles (Nsp)	Frequency of Species-Specific Profiles (f_{sp})	Number of Species-Shared Profiles	Number of Minimum Hypervariable Regions for Discrimination of All Species
Secale cereale ssp. *afghanicum*	4	2	1	0.73	0.54	4	1	0	1
Secale cereale ssp. *ancestrale*	16	6	4	2.02	0.78	16	1	0	2
Secale cereale ssp. *cereale*	16	7	5	2.56	0.71	16	1	0	2
Secale cereale ssp. *segetale*	16	6	4	2.10	0.69	16	1	0	2
Secale cereale ssp. *dighoricum*	8	4	1	0.88	0.71	8	1	0	1
Secale cereale ssp. *rigidum*	8	4	2	1.05	0.68	8	1	0	1
Secale strictum ssp. *africanum*	8	3	1	0.44	0.72	8	1	0	1
Secale strictum ssp. *anatolicum*	12	5	2	1.04	0.74	12	1	0	1
Secale strictum ssp. *kuprijanovii*	16	7	3	1.57	0.77	16	1	0	2
Secale strictum ssp *strictum*	12	5	4	2.65	0.67	12	1	0	3

Secale strictum ssp. *ciliatoglume*	4	2	1	0.61	0.59	4	1	0	1
Secale vavilovii	16	6	4	2.58	0.69	16	1	0	2
Secale sylvestre	16	7	6	2.92	0.55	16	1	0	4

Table S3. General description of standard SPInDel profiles from the *trnT*(UGU)-*trnL*(UAA)5′exon cpDNA region.

Species	Number of Sequences (N)	Number of Conserved Regions	Number of Hypervariable Regions (n)	Average Number of Pairwise Differences (p^{G}_{n})	Average Number of Pairwise Differences per Hypervariable Region	Number of Species-Specific Profiles (Nsp)	Frequency of Species-Specific Profiles (f_{sp})	Number of Species-Shared Profiles	Number of Minimum Hypervariable Regions for Discrimination of All Species
Secale cereale ssp. *afghanicum*	4	3	1	0.44	0.34	4	1	0	1
Secale cereale ssp. *ancestrale*	16	7	6	3.02	0.78	16	1	0	2
Secale cereale ssp. *cereale*	16	8	6	2.96	0.82	16	1	0	3
Secale cereale ssp. *segetale*	16	8	4	1.93	0.50	16	1	0	1
Secale cereale ssp. *dighoricum*	8	5	3	1.65	0.67	8	1	0	1
Secale cereale ssp. *rigidum*	8	4	2	1.11	0.57	8	1	0	1
Secale strictum ssp. *africanum*	8	5	2	0.99	0.61	8	1	0	1
Secale strictum ssp. *anatolicum*	12	6	4	1.92	0.64	12	1	0	2
Secale strictum ssp. *kuprijanovii*	16	9	7	2.97	0.79	16	1	0	3
Secale strictum ssp *strictum*	12	5	4	2.20	0.82	12	1	0	2

Secale strictum ssp. *ciliatoglume*	4	3	2	0.73	0.87	4	1	0	1
Secale vavilovii	16	7	5	2.54	0.67	16	1	0	3
Secale sylvestre	16	7	6	3.03	0.79	16	1	0	3

Table S4. General description of standard SPInDel profiles from the *trnL*(UAA) intron cpDNA region.

Species	Number of Sequences (N)	Number of Conserved Regions	Number of Hypervariable Regions (n)	Average Number of Pairwise Differences (p^{G}_{n})	Average Number of Pairwise Differences per Hypervariable Region	Number of Species-Specific Profiles (Nsp)	Frequency of Species-Specific Profiles (f_{sp})	Number of Species-Shared Profiles	Number of Minimum Hypervariable Regions for Discrimination of All Species
Secale cereale ssp, *afghanicum*	4	2	1	0.76	0.58	4	1	0	1
Secale cereale ssp, *ancestrale*	16	8	6	2.97	0.68	16	1	0	4
Secale cereale ssp, *cereale*	16	8	5	2.46	0.61	16	1	0	2
Secale cereale ssp, *segetale*	16	9	8	4.17	0.82	16	1	0	4
Secale cereale ssp, *dighoricum*	8	4	3	1.70	0.67	8	1	0	1
Secale cereale ssp, *rigidum*	8	4	2	1.01	0.58	8	1	0	1
Secale strictum ssp, *africanum*	8	5	4	1.89	0.84	8	1	0	1
Secale strictum ssp, *anatolicum*	12	7	5	2.25	0.80	12	1	0	2
Secale strictum ssp, *kuprijanovii*	16	9	7	4.02	0.91	16	1	0	4
Secale strictum ssp *strictum*	12	6	3	2.07	0.68	12	1	0	1

Secale strictum ssp, *ciliatoglume*	4	2	1	0.44	0.70	4	1	0	1
Secale vavilovii	16	8	5	2.61	0.66	16	1	0	3
Secale sylvestre	16	8	6	2.87	0.69	16	1	0	2

Table S5. General description of standard SPInDel profiles from the *trnD*[tRNA-Asp(GUC)]-*trnT*[tRNA-Thr(GGU)] cpDNA region.

Species	Number of Sequences (N)	Number of Conserved Regions	Number of Hypervariable Regions (n)	Average Number of Pairwise Differences (p^{G}_{n})	Average Number of Pairwise Differences per Hypervariable Region	Number of Species-Specific Profiles (Nsp)	Frequency of Species-Specific Profiles (f_{sp})	Number of Species-Shared Profiles	Number of Minimum Hypervariable Regions for Discrimination of All Species
Secale cereale ssp, *afghanicum*	4	3	1	0.78	0.66	4	1	0	1
Secale cereale ssp, *ancestrale*	16	7	5	2.58	0.67	16	1	0	2
Secale cereale ssp, *cereale*	16	8	7	3.47	0.71	16	1	0	2
Secale cereale ssp, *segetale*	16	7	6	2.81	0.60	16	1	0	3
Secale cereale ssp, *dighoricum*	8	3	2	0.76	0.31	8	1	0	1
Secale cereale ssp, *rigidum*	8	5	3	1.55	0.62	8	1	0	1
Secale strictum ssp, *africanum*	8	5	4	2.24	0.70	8	1	0	2
Secale strictum ssp, *anatolicum*	12	7	6	2.78	0.75	12	1	0	4
Secale strictum ssp, *kuprijanovii*	16	8	5	2.57	0.65	16	1	0	2
Secale strictum ssp *strictum*	12	5	3	1.73	0.48	12	1	0	1

Secale strictum ssp, *ciliatoglume*	4	3	1	0.44	0.62	4	1	0	1
Secale vavilovii	16	7	6	4.01	0.58	16	1	0	2
Secale sylvestre	16	8	5	2.66	0.62	16	1	0	2

Table S6. General description of standard SPInDel profiles from the *nad1*exon B-*nad1*exon C intron mtDNA region.

Species	Number of Sequences (N)	Number of Conserved Regions	Number of Hypervariable Regions (n)	AverageNnumber of Pairwise Differences (p^{G}_{n})	Average Number of Pairwise Differences per Hypervariable Region	Number of Species-Specific Profiles (Nsp)	Frequency of Species-Specific Profiles (f_{sp})	Number of Species-Shared Profiles	Number of Minimum Hypervariable Regions for Discrimination of All Species
Secale cereale ssp. *afghanicum*	6	4	3	1.52	0.44	6	1	0	1
Secale cereale ssp. *ancestrale*	24	11	9	4.55	0.76	24	1	0	5
Secale cereale ssp. *cereale*	24	9	8	4.08	0.79	24	1	0	6
Secale cereale ssp. *segetale*	24	11	7	4.01	0.72	24	1	0	4
Secale cereale ssp. *dighoricum*	12	5	4	2.10	0.65	12	1	0	2
Secale cereale ssp. *rigidum*	12	6	4	2.44	0.70	12	1	0	1
Secale strictum ssp. *africanum*	12	5	3	1.48	0.74	12	1	0	1
Secale strictum ssp. *anatolicum*	18	8	7	2.99	0.67	18	1	0	4
Secale strictum ssp. *kuprijanovii*	24	12	10	5.02	0.88	24	1	0	7
Secale strictum ssp *strictum*	18	7	6	2.81	0.65	18	1	0	4

Secale strictum ssp. *ciliatoglume*	6	3	2	1.07	0.27	6	1	0	1
Secale vavilovii	24	10	9	4.51	0.76	24	1	0	7
Secale sylvestre	24	11	8	4.04	0.69	24	1	0	5

Table S7. General description of standard SPInDel profiles from the *nad4/1-2* mtDNA region.

Species	Number of Sequences (N)	Number of Conserved Regions	Number of Hypervariable Regions (n)	Average Number of Pairwise Differences (p^{G}_{n})	Average Mumber of Pairwise Differences per Hypervariable Region	Number of Species-Specific Profiles (Nsp)	Frequency of Species-Specific Profiles (f_{sp})	Number of Species-Shared Profiles	Number of Minimum Hypervariable Regions for Discrimination of All Species
Secale cereale ssp. *afghanicum*	6	2	1	0.55	0.41	6	1	0	1
Secale cereale ssp. *ancestrale*	24	10	8	4.95	0.76	24	1	0	4
Secale cereale ssp. *cereale*	24	9	6	2.99	0.82	24	1	0	2
Secale cereale ssp. *segetale*	24	11	7	4.01	0.78	24	1	0	4
Secale cereale ssp. *dighoricum*	12	7	4	1.95	0.55	12	1	0	1
Secale cereale ssp. *rigidum*	12	5	3	1.56	0.60	12	1	0	1
Secale strictum ssp. *africanum*	12	5	4	2.01	0.49	12	1	0	2
Secale strictum ssp. *anatolicum*	18	8	6	2.97	0.44	18	1	0	4
Secale strictum ssp. *kuprijanovii*	24	10	6	2.94	0.47	24	1	0	3
Secale strictum ssp *strictum*	18	7	4	2.05	0.42	18	1	0	2

Secale strictum ssp. *ciliatoglume*	6	2	1	0.42	0.27	6	1	0	1
Secale vavilovii	24	11	7	3.50	0.52	24	1	0	5
Secale sylvestre	24	10	8	3.65	0.47	24	1	0	4

Table S8. General description of standard SPInDel profiles from the *nad4L-orf25* mtDNA region.

Species	Number of Sequences (N)	Number of Conserved Regions	Number of Hypervariable Regions (n)	Average Number of Pairwise Differences (p^{G}_{n})	Average Number of Pairwise Differences per Hypervariable Region	Number of Species-Specific Profiles (Nsp)	Frequency of Species-Specific Profiles (f_{sp})	Number of Species-Shared Profiles	Number of Minimum Hypervariable Regions for Discrimination of All Species
Secale cereale ssp. *afghanicum*	6	4	3	1.04	0.64	6	1	0	1
Secale cereale ssp. *ancestrale*	24	9	8	3.51	0.47	24	1	0	3
Secale cereale ssp. *cereale*	24	10	7	2.95	0.42	24	1	0	3
Secale cereale ssp. *segetale*	24	10	9	4.22	0.48	24	1	0	5
Secale cereale ssp. *dighoricum*	12	5	3	1.20	0.33	12	1	0	1
Secale cereale ssp. *rigidum*	12	6	5	2.67	0.50	12	1	0	3
Secale strictum ssp. *africanum*	12	6	3	1.73	0.56	12	1	0	1
Secale strictum ssp. *anatolicum*	18	8	6	2.75	0.48	18	1	0	3
Secale strictum ssp. *kuprijanovii*	24	9	7	3.18	0.43	24	1	0	3
Secale strictum ssp *strictum*	18	8	6	2.87	0.41	18	1	0	2

Secale strictum ssp. *ciliatoglume*	6	3	1	0.44	0.58	6	1	0	1
Secale vavilovii	24	9	8	3.87	0.43	24	1	0	3
Secale sylvestre	24	12	10	4.90	0.66	24	1	0	6

Table S9. General description of standard SPInDel profiles from the *rps12-1/nad3-2* mtDNA region.

Species	Number of Sequences (N)	Number of Conserved Regions	Number of Hypervariable Regions (n)	Average Number of Pairwise Differences (p^{G}_{n})	Average Number of Pairwise Differences per Hypervariable Region	Number of Species-Specific Profiles (Nsp)	Frequency of Species-Specific Profiles (f_{sp})	Number of Species-Shared Profiles	Number of Minimum Hypervariable Regions for Discrimination of All Species
Secale cereale ssp. *afghanicum*	6	4	3	1.24	0.75	6	1	0	2
Secale cereale ssp. *ancestrale*	24	9	7	3.20	0.48	24	1	0	3
Secale cereale ssp. *cereale*	24	11	10	5.01	0.47	24	1	0	6
Secale cereale ssp. *segetale*	24	10	6	2.78	0.42	24	1	0	2
Secale cereale ssp. *dighoricum*	12	5	4	1.97	0.34	12	1	0	1
Secale cereale ssp. *rigidum*	12	5	3	1.22	0.38	12	1	0	1
Secale strictum ssp. *africanum*	12	6	5	2.45	0.51	12	1	0	3
Secale strictum ssp. *anatolicum*	18	8	6	2.76	0.67	18	1	0	2
Secale strictum ssp. *kuprijanovii*	24	12	10	5.02	0.86	24	1	0	5
Secale strictum ssp *strictum*	18	8	6	2.65	0.63	18	1	0	2

Secale strictum ssp. *ciliatoglume*	6	3	1	0.48	0.50	6	1	0	1
Secale vavilovii	24	10	8	3.97	0.67	24	1	0	4
Secale sylvestre	24	11	7	3.01	0.77	24	1	0	4

Table S10. General description of standard SPInDel profiles from the *rps12-2/nad3-1* mtDNA region.

Species	Number of Sequences (N)	Number of Conserved Regions	Number of Hypervariable Regions (n)	Average Number of Pairwise Differences (p^{G}_{n})	Average Mumber of Pairwise Differences per Hypervariable Region	Number of Species-Specific Profiles (Nsp)	Frequency of Species-Specific Profiles (f_{sp})	Number of Species-Shared Profiles	Number of Minimum Hypervariable Regions for Discrimination of All Species
Secale cereale ssp. *afghanicum*	6	4	3	1.45	0.82	6	1	0	2
Secale cereale ssp. *ancestrale*	24	12	10	5.54	0.79	24	1	0	5
Secale cereale ssp. *cereale*	24	8	6	3.02	0.44	24	1	0	3
Secale cereale ssp. *segetale*	24	10	8	4.25	0.48	24	1	0	3
Secale cereale ssp. *dighoricum*	12	5	4	2.43	0.43	12	1	0	1
Secale cereale ssp. *rigidum*	12	6	4	2.17	0.59	12	1	0	2
Secale strictum ssp. *africanum*	12	7	5	2.40	0.73	12	1	0	3
Secale strictum ssp. *anatolicum*	18	8	6	2.99	0.45	18	1	0	3
Secale strictum ssp. *kuprijanovii*	24	11	7	3.60	0.49	24	1	0	4
Secale strictum ssp *strictum*	18	8	5	2.76	0.55	18	1	0	2

Secale strictum ssp. *ciliatoglume*	6	4	3	1.45	0.87	6	1	0	1
Secale vavilovii	24	12	10	4.91	0.78	24	1	0	4
Secale sylvestre	24	9	6	2.67	0.51	24	1	0	4

Table S11. General description of standard SPInDel profiles from the *rrn5*/*rrn18-1* mtDNA region.

Species	Number of Sequences (N)	Number of Conserved Regions	Number of Hypervariable Regions (n)	Average Number of Pairwise Differences (p^{G}_{n})	Average Number of Pairwise Differences per Hypervariable Region	Number of Species-Specific Profiles (Nsp)	Frequency of Species-Specific Profiles (f_{sp})	Number of Species-Shared Profiles	Number of Minimum Hypervariable Regions for Discrimination of All Species
Secale cereale ssp. *afghanicum*	6	4	3	0.59	0.89	6	1	0	1
Secale cereale ssp. *ancestrale*	24	10	8	3.89	0.45	24	1	0	4
Secale cereale ssp. *cereale*	24	11	8	4.12	0.49	24	1	0	5
Secale cereale ssp. *segetale*	24	10	9	4.48	0.42	24	1	0	5
Secale cereale ssp. *dighoricum*	12	5	4	2.02	0.56	12	1	0	2
Secale cereale ssp. *rigidum*	12	6	4	1.97	0.61	12	1	0	1
Secale strictum ssp. *africanum*	12	7	5	2.47	0.78	12	1	0	2
Secale strictum ssp. *anatolicum*	18	9	7	3.88	0.81	18	1	0	4
Secale strictum ssp. *kuprijanovii*	24	11	8	4.04	0.60	24	1	0	4
Secale strictum ssp *strictum*	18	9	5	2.57	0.73	18	1	0	3

Secale strictum ssp. *ciliatoglume*	6	3	2	0.96	0.77	6	1	0	1
Secale vavilovii	24	11	10	5.16	0.62	24	1	0	5
Secale sylvestre	24	10	6	2.77	0.55	24	1	0	4
